# Prevalence and risk factors of anemia in the mother–child population from a region of the Colombian Caribbean

**DOI:** 10.1186/s12889-023-16475-0

**Published:** 2023-08-12

**Authors:** Lisetta Del Castillo, Nora Cardona-Castro, Denis R. Whelan, John Paul Builes, Héctor Serrano-Coll, Margarita Arboleda, Juan S. Leon

**Affiliations:** 1 Hubert Department of Global Health, Emory University. Atlanta, Georgia, USA; 2 Instituto Colombiano de Medicina Tropical, Universidad CES, Medellín, Colombia; 3 Biostatistics and Bioinformatics, Emory University. Atlanta, Georgia, USA

**Keywords:** Public health, Surveillance, Poverty, Anemia

## Abstract

**Background:**

Despite Colombia's robust well-child visits program, Colombian children and mothers still suffer from anemia, especially in populations of lower socioeconomic status. In this study, we aimed to quantify the prevalence and risk factors among mothers and their children attending their well-child visits in Apartadó, a municipality in the Urabá region of the Colombian Caribbean.

**Methods:**

There were 100 mother–child pairs enrolled in this secondary data-analysis study from a health facility in the municipality of Apartadó, Urabá, Colombia, during well-child visits. Self-reported data included child illnesses in the past two weeks (diarrheal, fever, or respiratory symptoms), child feeding practices (breastfeeding, complementary feeding), child vaccinations, and demographic characteristics (mother’s and child’s age, mother’s education, marital status, race, and child sex) and socioeconomic status. Mother and child anthropometry data were collected via standardized weight and height measurements. Mother or child anemia status was collected via a blood test. Chi-squared tests and multivariable logistic regression were used to assess associations between risk factors and anemia.

**Result:**

The anemia prevalence in children (74%) and mothers (47%) was higher than the Colombian national prevalence. Reported child comorbidities in the preceding two weeks were not significantly associated with child anemia and included respiratory illnesses (60%), fever (46%), and diarrhea (30%). Stunting (8%) was not significantly associated with anemia. Wasting (0%) was not observed in this study. Reported child breastfeeding and complementary feeding were also not significantly associated with child anemia. In adjusted models, the child's significant risk factors for anemia included the mother's "Mestiza" race (OR: 4.681; 95% CI: 1.258, 17.421) versus the Afro-Colombian race. Older children (25–60 months) were less likely to develop anemia than younger (6–24 months) children (OR: 0.073; 95% CI: 0.015, 0.360).

**Conclusions:**

The finding of high anemia prevalence in this study advances our understanding of child and maternal anemia in populations of low socioeconomic status where health care is regularly accessed through well-child programs.

**Supplementary Information:**

The online version contains supplementary material available at 10.1186/s12889-023-16475-0.

## Background

Globally, 30% of women and 40% of children under five years old have anemia [1]. This disease has a multifactorial etiology. Iron deficiency is the most common cause of anemia in developing countries. Anemia is associated with infections and infant mortality deaths worldwide.

In countries such as Colombia, anemia is often linked to poverty stemming from poor nutrition [2]. Although Colombia is classified as a middle-income country, in 2014, 29% of Colombians were poor, and 6 million people lived below the poverty line of $3.50 (USD) per day [3]. Poverty is higher in rural regions, and according to the National Survey of the Nutritional Situation in Colombia [4], impoverished people suffering from anemia do not have the necessary resources to buy iron-rich food consistently for themselves and their families. Approximately 43% of Colombians living in rural regions considered themselves food insecure due to poverty [5]. Additionally, studies examining the implications of food insecurity revealed that the absence of iron nutrients impacts cognitive development and impairs productivity levels, condemning people to a vicious cycle of poverty [2]. Despite Colombia’s robust well-child visits program, children’s and mothers’ anemia status, in populations of lower socio-economic status, may not be well captured or addressed. In this study, we aimed to quantify the prevalence and risk factors for anemia in a mother–child, mixed ancestry population attending their well-child visits in a region of the Colombian Caribbean, Apartadó. The findings from this study advance our understanding of child and maternal anemia, in populations with a lower socioeconomic status, where health care is regularly accessed through well-child programs. The study may also provide a valuable comparison group for similar populations in other world regions.

## Methods

### Study design, location, population, and approval

**Fig. 1:**
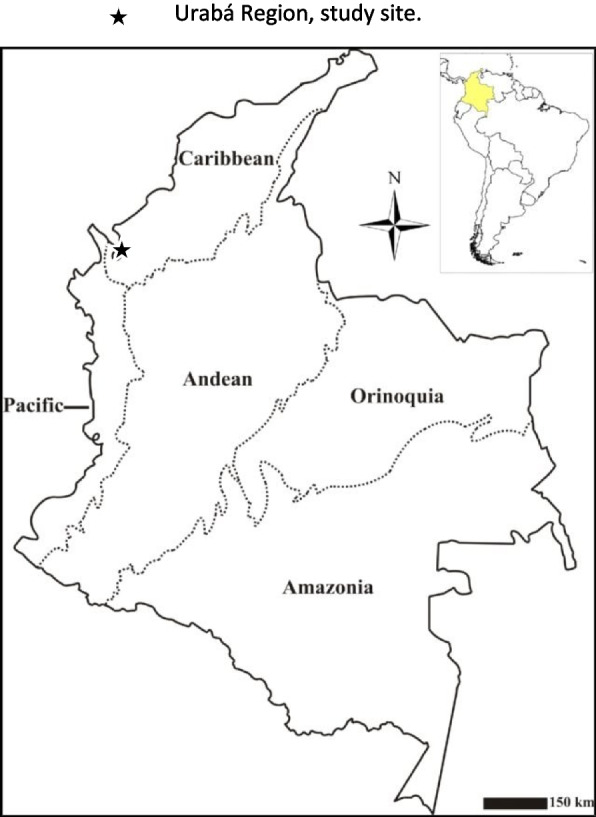
Study site

This is a secondary data-analysis based on a subsample of a cross-sectional observational study designed to measure the prevalence of stunting of children attending their well-child visits in Apartadó, Colombia. The municipality of Apartadó is in the Caribbean region of Urabá. The region of Urabá is located in northwestern Colombia near the Panama border (Fig. 1) at an average altitude of 30 m above sea level; in 2016, it had a population of 676,356 people [6]. The design of the original study has been described in more detail [7]. In brief, 198 mothers and children between 6 and 60 months of age were recruited, between June and July of 2016, in the waiting room during their well-child visit to the health facility in Apartadó while waiting for their appointments with health professionals. The inclusion criteria for mother–child participation included the following: 1) volunteer mothers of at least 15 years old with fluency in Spanish; and 2) a healthy child aged 6 to 60 months old registered to attend well-child visits. If the mother met the inclusion criteria and verbally expressed interest in participating in the study, the informed consent process was carried out verbally and in writing and documented. Mothers younger than 18 signed an assent form according to ethical rules for minors. Both Emory University (00088473) and Universidad CES IRBs (session 85, 14 September 2015) approved the original study.

### Data entry and data quality of the original study

In the original study, data quality was ensured through double data entry of the paper-based survey data to an electronic database by two trained study staff. Statistical Analysis Software (SAS) v9.4 (Cary, NC, USA) was used to compare both databases to address discrepancies related to duplicate entries, missing values, or data errors. Discrepancies were addressed using the original survey forms to verify that correct inputs and corrections were made in the original database. The reconciliation process was documented in a monitoring log to ensure data quality. Additionally, standard diagnostic procedures such as checking for outliers in the data graphs were used to detect data errors.

### Sample size of the secondary data-analysis study on child anemia

For this secondary data-analysis study on child anemia, to estimate the sample size [8] needed to measure the prevalence of anemia among children in the original’s study data set, we assumed an alpha level of 0.05, precision of 7%, and an 11.3% prevalence of anemia among children under 11 years old from Urabá [9]. The calculated sample size required was 79 children. In the original’s study data set (198 mother children pairs), 100 children had both anemia data and child-related and demographic-related characteristics. These 100 children were the sample used in this secondary data-analysis study.

### Data collection tools & study activities

The illness, demographic, and socioeconomic data used in this study were collected in the original study using pretested and structured health surveys through interviews with mothers. The data used included mothers’ self-reported information about themselves and their children with regard to illnesses (e.g., respiratory, fever, or diarrheal symptoms in the past 2 weeks), frequency of breastfeeding, feeding practices (e.g., complementary feeding), vaccination coverage (photographs of vaccine cards for data entry), household conditions, self-reported demographic characteristics (e.g., mother’s and child’s age, mother’s education, marital status, race, and child sex), and socioeconomic status. Socioeconomic status was noted using Colombia’s social stratification system ranging from 1–6, with 1 being the poorest and 6 being the richest. Levels 1 (low-low), 2 (low), and 3 (low-medium) are generally classified as being in the lower strata [10]. The Colombian socioeconomic strata are based on household and neighborhood conditions and not income.

The anthropometry data used in this study was measured in the original study by a two-person team of trained interviewers whose measurements were validated with a non-study reference population before study initiation. Height and weight were measured to identify height-for-age, weight-for-age, and weight-for-height anthropometric parameters using a digital SECA scale and a Shorrboard® [11]. Additionally, individual Z scores were calculated using Anthro software, developed by the World Health Organization (WHO). Weight-for-height, height-for-age and weight-for-age were interpreted by using the Z score classification system. A Z score below 2 indicated that there was a health condition affecting the growth of the child, including undernutrition, stunting, and wasting. Moreover, in the mother, height and weight measures were used to calculate body mass index (BMI) based on guidelines from the National Institute of Health to identify the mother’s health conditions.

The anemia data used in this study was measured in the original study using HemoCue® to determine the level of hemoglobin from a single drop of blood from a finger prick of the mother or child. According to WHO guidelines for anemia, pregnant mothers and children aged 6 to 60 months who have hemoglobin levels < 11 g/dL are considered anemic; those with levels of 0.0–10.9 g/dL are considered mildly anemic, those with levels of 7–9.9 g/dL are considered moderately anemic, and those with levels < 7 g/dL are considered severely anemic. The cutoff point for anemia among nonpregnant women is a hemoglobin level of < 12 g/dL [12].

In the original study, at the end of the procedure, the mother was given a form with the results of her hemoglobin test and BMI assessment as well as the values of hemoglobin and anthropometric parameters of her child. A referral to see a doctor was given to mothers and children with hemoglobin levels below their respective cutoff points. Referrals were also provided for children who had Z score values less than 2 and for mothers with BMI values greater than 25. Additional counseling by the study team was provided to encourage healthy eating habits and exercise. Upon the completion of the study activities, the team provided the mother and child with a small gift to express gratitude for their participation in the study.

### Data analysis

In this study, statistical Analysis Software (SAS) v9.4 (Cary, NC, USA) was used for all analyses. Descriptive analysis was used to characterize the sociodemographic and health characteristics of the study population. Bivariate analyses between clinical characteristics and child sex or between mother’s anemia, BMI, and mother’s age were performed by applying a chi-squared analysis. Multivariable analysis to identify child risk factors for anemia was performed by applying a logistic regression model. In the logistic regression model, the dependent variable was child anemia status. Independent variables included child illness and child feeding practices. An evaluation of confounding (10% rule) was performed [13, 14] on covariates thought to be associated with child anemia from the published literature, including child-related, maternal-related, and demographic-related characteristics [15–18]. Unadjusted and adjusted odds ratios (ORs) and 95% confidence intervals (CIs) were reported for the association between anemia and risk factors. An alpha level of *p* < 0.05 was considered significant.

## Results

### Sociodemographic characteristics of the mother–child pair

This secondary data-analysis study on child anemia evaluated children between 6 and 60 months and mothers aged 15 to 46 years old. Close to three-quarters of the children were under two years old, and one-quarter of the children were between the ages of two and five years old (Table 1). Close to half of children were reported to be female. More than half of the mothers were between the ages of 15 and 24. Close to three quarters of mothers reported attending any level of high school, and 14 percent of mothers reported attending college. The majority of mothers reported cohabitating with a partner or being married. Fifty-one percent of the mothers reported being of the Mestiza race, 36 percent of the mothers reported being of the Afro-Colombian race, 10 percent of the mothers reported being of the White race, and 3 percent of the mothers reported being of the Indigenous race. All mothers reported being of low socioeconomic status, with 79% of mothers reporting being in the lowest strata. Additionally, despite the challenging living conditions related to poverty, 84 percent of the mothers reported having access to safe water connections in their households (data not shown). The remainder reported accessing water from streams, rivers, or creeks (7 percent), a manually operated (4 percent) or mechanical (2 percent) well pump, or a water connection outside the house and other answers (3 percent) (data not shown).

**Table 1:** Socio-demographic characteristics of the mother–child pair

Socio-Demographic Characteristic	N (%)
**Child’s age (month)**	
6–24 months	74 (74)
25—60 months	26 (26)
**Child’s sex**	
Female	46 (46)
Male	54 (54)
**Mother’s age (years)**	
15 – 24	52 (52)
25 – 34	30 (30)
35 – 46	18 (18)
**Mother’s education level**	
Any Primary School	12 (12)
Any High School	74 (74)
College or Higher Education	14 (14)
**Mother’s marital status**	
Cohabitating or Married	61 (61)
Single	39 (39)
**Mother’s race**	
Afro-Colombian	36 (36)
Indigenous	3 (3)
Mestiza (Mixed-Race)	51 (51)
White	10 (10)
**Mother’s Colombian socio-economic classification**	
Strata 1 (low-low)	79 (79)
Strata 2 (low)	20 (20)
Strata 3 (low-medium)	1 (1)

As a proxy for access to care at these well-child visits, we also examined whether children were fully vaccinated (received all their age-appropriate vaccine doses, Table 1S-Supplementary Material). Children were fully vaccinated with Bacillus Calmette–Guérin (BCG, 99 percent), hepatitis B (97 percent), pneumococcus (97 percent) and rotavirus (97 percent). Children were also fully vaccinated with MMR (88%), polio (88 percent), the pentavalent vaccine (85 percent), and hepatitis A (81 percent). The vaccines with the lowest number of children fully vaccinated were seasonal influenza (68 percent) and yellow fever (60 percent). A level of at least 60 percent of children being fully vaccinated according to age was achieved by all vaccines.

### Clinical characteristics of children

Seventy-four percent of children in the study were anemic (Table 2). Given that past illnesses [19], anthropometry status, or other clinical characteristics may provide hints as to overall nutritional status influencing anemia, we also analyzed these factors. As reported by their mothers, in the preceding two weeks, 30% of children in the study experienced diarrhea episodes, and 46% experienced fever symptoms. Mothers reported that 60 percent of children experienced respiratory illnesses. Unlike other health conditions, stunting (8 percent) and wasting (0 percent) were not predominantly prevalent in the study population. Males (13 percent), compared to females (2 percent), had a substantially higher prevalence of stunting. No other clinical characteristic significantly differed by child’s sex.

**Table 2:** Health characteristics of children (6–60 months)

	**Female**^a^	**Male**^a^	**All**^a^
Anemia (< 11 Hemoglobin)	33 (72)	41 (76)	74 (74)
Diarrhea	11 (24)	19 (35)	30 (30)
Fever (> 37 Celsius)	21 (46)	25 (46)	46 (46)
Respiratory Diseases	26 (57)	34 (63)	60 (60)
Stunting (< 2 Standard Deviations)	1 (2)	7 (13)^b^	8 (8)
Wasting (< 2 Standard Deviations)	0 (0)	0 (0)	0 (0)

^a^N (% with that characteristic among the total population of female, male, or all)

^b^Significantly higher based on a chi square test; alpha level of 0.05

To assess whether any of these clinical characteristics were associated with child anemia, we analyzed the relationship of child anemia with measures of nutritional status (stunting, wasting) and past illnesses and adjusted for demographic confounders (child sex, child age) (Table 3). No measure of nutritional status (stunting, wasting) or measures of illnesses (diarrhea, fever, respiratory diseases, in the preceding two weeks) were significantly associated with child anemia. In the unadjusted and adjusted models, older children (25–60 months) were substantially less likely to be anemic than younger children (6–24 months). Older children, compared to younger children, had a significantly lower prevalence of anemia (25–60 months: 12/26 [46%]; 6–24 months 62/74 [84%]). We did not observe significant differences in other clinical characteristics by child age.

**Table 3:** Relationship between health characteristics of children and anemia

	**Unadjusted**^a^		**Adjusted**^a^	
	**OR**	**95% Confidence Intervals**	**OR**	**95% Confidence Intervals**
Diarrhea (Yes/ No [Reference])	2.142	0.722, 6.359	1.355	0.363, 5.055
Fever (> 37 Celsius) (Yes/ No [Reference])	1.515	0.608, 3.773	1.388	0.399, 4.827
Respiratory Diseases (Yes/ No [Reference])	0.734	0.289, 1.862	0.514	0.154, 1.709
Stunting (< 2 Standard Deviations) (Yes/ No [Reference])	2.611	0.305, 22.312	2.575	0.259, 25.572
Child's Age (25–60 months/ 6–24 months [Reference])	0.165^b^	0.061, 0.445	0.178^b^	0.062, 0.508
Child's Sex (Male/ Female [Reference])	1.242	0.507, 3.040	1.013	0.365, 2.807

^a^Analysis did not include wasting because no wasting was detected in this dataset

^b^Significant association based on an alpha level of 0.05

We then evaluated additional child-related, maternal-related, and demographic characteristics [15, 16] as potential confounders of the relationship between child clinical characteristics and child anemia. Child-related characteristics included child sex and child age. Maternal-related characteristics included mother’s anemia and BMI status. Demographic-related characteristics included mother’s age, education, marital status, race, and socioeconomic condition. Logistic regression was used to evaluate the association between child clinical characteristics (diarrhea, fever, respiratory diseases, stunting, and wasting) with child anemia and was run with and without each potential confounding variable. All confounding variables modified the odds ratio of at least one clinical characteristic by more than 10% [13, 14] (data not shown). However, no confounding variable, whether singly or together, resulted in a child clinical characteristic becoming significantly associated with child anemia. Thus, to maximize sample size while controlling for relevant confounders, we chose to report the unadjusted and adjusted odds ratios between child clinical characteristics and child anemia while controlling for the most immediate confounders (child sex, child age), as reported in Table 3.

### Clinical characteristics of mothers

In this study, 47% of the 72 mothers who consented to testing for hemoglobin had anemia (Table 4). When stratified by age, mother’s anemia ranged between 46 and 50 percent. By chi-squared test, there was no significant relationship between the mother’s age and anemia status. Mother BMI was also assessed in 98 mothers with BMI data. Approximately 43 percent of the mothers had a normal weight, while 8 percent were underweight, 28 percent were overweight and 21 percent were obese. By chi-squared test, there was a significant association between BMI and age (*p* = 0.005). Generally, those of higher age had a higher BMI. Although there was a trend of decreasing anemia prevalence with increasing BMI (anemia prevalence: underweight = 5/7 [71%], normal weight = 17/30 [57%], overweight = 7/20 [35%], obesity = 4/14 [29%]), we do not report statistical significance due to low sample size.

**Table 4:** Mother health characteristics

	**Age in years. N (column %)**			
	15 – 24	25 – 34	35 – 46	Total
**Anemia status**				
Yes	17 (46)	10 (50)	7 (47)	34 (47)
No	20 (54)	10 (50)	8 (53)	38 (53)
Total	37	20	15	72
**Body mass index**^a^				
Underweight (< 18.5)	6 (12)	1 (3)	1 (6)	8 (8)
Normal weight (18.5—24.9)	26 (51)	14 (47)	2 (12)	42 (43)
Overweight (25—29.9)	9 (18)	11 (37)	7 (41)	27 (28)
Obesity (> 30)	10 (20)	4 (13)	7 (41)	21 (21)
Total	51	30	17	98

^a^Body mass index and Age were significantly associated based on a chi square test; alpha level of 0.05

### Risk factors associated with anemia

Given that neither markers of nutritional status (stunting, wasting) nor illnesses (diarrhea, fever, respiratory diseases, in the preceding two weeks) were significantly associated with child anemia, we then considered whether child feeding practices might be associated with child anemia [20]. We assessed the association of child anemia with two self-reported child feeding practices: complementary feeding of the child or any breastfeeding of the child. Similar to Table 3, in the unadjusted and adjusted models (Table 5), older children (25–60 months) were significantly less likely to be anemic than younger children (6–24 months) (adjusted OR 0.073; 95% CI 0.015, 0.360). We also found that in the unadjusted and adjusted models (Table 5), children from a mother of “Mestiza” race, compared to “Afro-Colombian” race, were significantly more likely to be anemic (adjusted OR 4.681; 95% CI 1.258, 17.421). Child anemia by mothers' race included Indigenous (3/3 [100%], Mestiza (43/51 [84%], Afro-Colombian (22/36 [61%]), and White (6/10 [60%]). In a subset analysis of complete records with mother’s anemia and BMI status (*n* = 69), where we also controlled for both maternal-related characteristics (mother’s anemia and BMI status), all Table 5 effect estimates were similar: child’s age was still significant, but “Mestiza” race was no longer significant, likely due to lower power (data not shown). In all models, complementary feeding or any breastfeeding of the child was not significantly associated with child anemia.

**Table 5:** Child feeding and child- and demographic-related factors associated with anemia

	**Unadjusted**^a^		**Adjusted**^a^	
	**OR**	**95% Confidence Intervals**	**OR**	**95% Confidence Intervals**
Complementary feeding (Yes/No [Reference])	0.795	0.153, 4.107	2.455	0.326, 18.455
Breastfeeding (Any/No [Reference])	1.750	0.686, 4.463	0.768	0.164, 3.599
Mother's Age (continuous)	0.966	0.911, 1.025	0.987	0.909, 1.072
Mother’s Education (College or Higher Education/ Any Primary School [Reference])	4.999	0.740, 33.776	4.914	0.450, 53.588
Mother’s Education (Any High School / Any Primary School [Reference])	2.453	0.667, 9.018	0.544	0.075, 3.896
Mother’s Marital Status (Cohabitating or Married / Single [Reference])	0.533	0.212, 1.339	0.446	0.141, 1.408
Mother's Race ("Mestiza”/ “Afro-Colombian" [Reference])	3.909^b^	1.378, 11.089	4.681^b^	1.258, 17.421
Mother's Race ("White”/ “Afro-Colombian" [Reference])	0.954	0.228, 3.995	1.214	0.171, 8.616
Mother’s Socio-Economic Status (2/1 [Reference])	2.308	0.614, 8.674	3.048	0.522, 17.791
Child's Sex (Male/Female [Reference])	1.320	0.529, 3.291	1.187	0.351, 4.014
Child’s Age (25–60 months/6–24 months [Reference])	0.159^b^	0.058, 0.436	0.073^b^	0.015, 0.360

^a^Mother’s Socio Economic Status of 3 (*n* = 1) and Race of “Indigenous” (*n* = 3) were excluded due to their small sample sizes that contributed to model instability. Final model had a sample size of 96 children

^b^Significant association based on an alpha level of 0.05

## Discussion

The high rate of anemia in this population (74 percent of children, 47 percent of mothers) is concerning given that it is higher than that stated in various reports on Colombia’s national anemia prevalence. For example, the 2010 Ministry of Health reported 33.2 percent anemia for children and 32.8 percent anemia for mothers [4] and the 2010 Demographic Health Surveys and National Nutrition Survey reported 27.5 percent anemia for children and 6.6–10.3 percent anemia for mothers [21, 22]. This high rate of anemia was also higher than that reported in a 2013 study of a nearby municipality (Turbo, Urabá) of 48.6% in children under 7 years of age [23]. However, our data are similar to the findings reported by Dos Santos et al. in Recife, Brazil, who observed a high prevalence of anemia in their pediatric population (56.6 percent) [24]. According to the WHO guidelines on the prevention and control of anemia, the prevalence of anemia globally should be less than 5%, and anemia is defined as a severe public health problem when anemia is higher than 40% [25]. Therefore, by definition, this region has a public health emergency. High rates of anemia in the region could be the result of socioeconomic barriers preventing individuals from accessing nutritious diets for healthy lifestyles [4].

Several studies have demonstrated that poverty, violence, and lack of education are all leading factors contributing to the risk of anemia in Colombia [2, 4, 5]. In Colombia, impoverished individuals suffering from anemia reported not having the necessary resources to buy iron-rich food [4]. Additionally, the armed conflict in Colombia forced millions of people to flee their homes, including in this region, leading to hunger emergencies and severe poverty, as the people who were displaced were left without any means to fend for themselves and their families [5, 26]. Last, high parasite prevalence in other Latin American regions has been linked with high anemia prevalence [27, 28]. This region’s children have exhibited high levels of both protozoal (e.g., 34 percent *Giardia lamblia*) and helminth infection (e.g., 44.5 percent Ascaris) in past studies [23]. Therefore, it is likely that individuals living in this tropical region are vulnerable to anemia and other health conditions due to precarious living conditions.

The data analysis suggested that younger children between the ages of six months and two years old had a higher risk of anemia than older children. It is possible that younger children are at an increased risk of anemia than older children because younger children are more likely to exclusively breastfeed than older children. Exclusively breastfed children do not benefit from the various sources of iron-rich foods, whereas older children are more likely to consume various sources of iron-rich foods, making them less susceptible to anemia. Most cross-sectional, cohort and clinical trials observed that iron deficiency anemia in young children was associated with exclusive and prolonged breastfeeding practices [29–32]. However, our findings did not show an effect of complementary feeding or breastfeeding on anemia prevalence. Another important factor that could be related to anemia in young children is the high rate of malaria in this region (35 cases × 1,000 exposed) [33], which would be related to hemolysis and anemia in susceptible age groups [33, 34] such as represented by the younger populations of this Caribbean region.

As mentioned, the study did not find a relationship between complementary feeding and anemia. The study’s findings are in line with existing evidence indicating that complementary feeding is not a risk factor for anemia [35–37]. To evaluate the risk of complementary feeding on iron status, prior clinical trials randomly assigned children to either continue exclusive breastfeeding or to receive iron-fortified foods in addition to breast milk [35, 36]. Based on follow-up data, complementally fed children had higher hemoglobin levels than exclusively breastfed children, and no significant correlation was found between complementally fed children and iron deficiency anemia [35, 36]. Furthermore, systematic studies on randomized controlled trials found no significant risk of anemia in complementary fed children [37, 38].

Causes of anemia include low hemoglobin and low numbers of red blood cells, leading to impaired oxygen delivery throughout the body [18]. There are many immediate factors that influence hemoglobin and red blood cell concentrations. These include infections (e.g., malaria) and poor diet leading to macronutrient deficiencies (exemplified by stunting and wasting) and micronutrient deficiencies (exemplified by low iron concentrations) [18]. These immediate factors may in turn be affected by distal factors (e.g., environmental conditions affecting risk of malaria or poor diet), and these distal factors may themselves be associated with anemia (i.e., confounders). Confounders of child anemia, from published studies, include child-, maternal-, and demographic-related characteristics [15–18]. Recent child illnesses may affect inflammation, nutrient absorption, and diet [39]. In our study, we did not find an association between recent child illnesses and child anemia. We did find that younger age (under 24 months) was significantly associated with anemia. A younger age may generally be associated with vulnerability to multiple illnesses, including anemia, due to dependence on the mother’s diet and feeding practices (e.g., breastfeeding, complementary feeding). We also found that race was associated with children’s anemia, even after adjusting for other confounders (socioeconomic status, education), similar to other findings [18]. It is possible that contextual factors, such as the ability and access to purchase nutrient-rich foods for the household or food feeding and preparation practices, may be associated with a mother’s race in the study region.

One strength of this study was the ability to work with individuals in this understudied population and begin to understand their health needs and health risk factors, especially those accessing health care through a well-child visits program. A limitation was the relatively small sample size of this cross-sectional study, which may have reduced our sensitivity to identify additional statistical relationships. A second limitation was that data specifying rural or urban residence, as potential demographic confounders of child anemia [16], were not collected. A third limitation was our inability, due to limited funding, to include serum nutritional biomarkers (e.g., ferritin, inflammatory biomarkers) to better understand the mechanisms of anemia in children and mothers.

## Conclusions

In this mixed ancestry population of low socioeconomic status, anemia rates were high in both mothers and children despite their positive health-access behaviors and their attendance at well-child visits. Additional studies are warranted in other similar populations attending well-child visits to assess anemia prevalence and identify possible preventative or treatment strategies.

### Supplementary Information

**Additional file 1: Table 1S.** Vaccination Status According to doses and Vaccines.

## Data Availability

The datasets used and/or analyzed during the current study are available from the corresponding author upon reasonable request.
