# Supplementary material for: Prevalence and risk factors of anemia in the mother–child population from a region of the Colombian Caribbean
Source: BMC Public Health. 2023 Aug 12;23:1533. doi: 10.1186/s12889-023-16475-0 (PMC10422807; doi:10.1186/s12889-023-16475-0)
Supplement: Supplementary file 1 — Additional file 1: Table 1S. Vaccination Status According to doses and Vaccines. [file 12889_2023_16475_MOESM1_ESM.docx]

**Table 1S. Vaccination Status According to doses and Vaccines**

| **Variable*** | **BCG** | | **Penta**  **valent** | | **Hepatitis B** | | **Polio** | | | **Rota**  **virus** | | **Pneumococcus** | | **Seasonal influenza** | | | **MMR** | | | **Hepatitis A** | | | **Yellow Fever** | | |
| --- | --- | --- | --- | --- | --- | --- | --- | --- | --- | --- | --- | --- | --- | --- | --- | --- | --- | --- | --- | --- | --- | --- | --- | --- | --- |
|  | n | % | n | % | n | % | | n | % | n | % | n | % | n | % | n | | % | n | | % | n | | % |  |
| Received the 1st dose | 155 | 99 | 156 | 100 | 151 | 97 | | 156 | 100 | 154 | 99 | 157 | 100 | 115 | 74 | 87 | | 88 | 77 | | 81 | 59 | | 60 |  |
| Received the 2nd dose | - | - | 154 | 98 | - | - | | 154 | 99 | 152 | 97 | 153 | 98 | 91 | 68 | - | | - | - | | - | - | | - |  |
| Received the 3rd dose | - | - | 134 | 86 | - | - | | 136 | 88 | - | - | - | - | - | - | - | | - | - | | - | - | | - |  |
| Received the 1st booster** | - | - | - | - | - | - | | 57 | 85 | - | - | 84 | 85 | - | - | + | | + | - | | - | - | | - |  |
| Received the 2nd booster** | - | - | - | - | - | - | | + | + | - | - | - | - | - | - | - | | - | - | | - | - | | - |  |
| Under vaccinated*** | 1 | 1 | 24 | 15 | 5 | 3 | | 19 | 12 | 4 | 3 | 4 | 3 | 42 | 32 | 12 | | 12 | 18 | | 19 | 40 | | 40 |  |
| Fully vaccinated**** | 155 | 99 | 133 | 85 | 151 | 97 | | 136 | 88 | 152 | 97 | 153 | 97 | 91 | 68 | 87 | | 88 | 77 | | 81 | 59 | | 60 |  |

* The symbol “-” indicates that there were no data available for that entry

** The symbol “+” indicates that no children eligible for the vaccine received the vaccine

*** Under vaccinated was defined as children who did not receive all of the doses of the vaccine scheduled for their age group excluding the booster shots for Polio, Pneumococcus, and MMR vaccines

**** Fully vaccinated was defined as children who received each dose of the vaccine that they were eligible to receive for their age. Vaccines do not include the booster shots for the Polio, Pneumococcus, and MMR vaccines
